# Revitalizing Fertility of Nutrient-Deficient Virgin Sandy Soil Using Leguminous Biocompost Boosts *Phaseolus vulgaris* Performance

**DOI:** 10.3390/plants10081637

**Published:** 2021-08-10

**Authors:** Mahmoud A. Abdelfattah, Mostafa M. Rady, Hussein E. E. Belal, Eman E. Belal, Rahmah Al-Qthanin, Hatim M. Al-Yasi, Esmat F. Ali

**Affiliations:** 1 Soils and Water Science Department, Faculty of Agriculture, Fayoum University, Fayoum 63514, Egypt; eeb00@fayoum.edu.eg; 2 Food and Agriculture Organization of the United Nations (FAO), Cairo 11668, Egypt; 3 Botany Department, Faculty of Agriculture, Fayoum University, Fayoum 63514, Egypt; hes00@fayoum.edu.eg; 4 Prince Sultan Bin-Abdul-Aziz Center for Environment and Tourism Studies and Researches, King Khalid University, P.O. Box 960, Abha 61421, Saudi Arabia; alqthanin-r@hotmail.com; 5 School of Biological Sciences, King Khalid University, P.O. Box 960, Abha 61421, Saudi Arabia; 6 Department of Biology, College of Science, Taif University, P.O. Box 11099, Taif 21944, Saudi Arabia; h.alyasi@tu.edu.sa (H.M.A.-Y.); a.esmat@tu.edu.sa (E.F.A.)

**Keywords:** virgin soils, bio-organic and chemical fertilizer, soil properties, *Phaseolus vulgaris*

## Abstract

During the 2019 and 2020 seasons, nutrient-deficient virgin sandy soil was examined along with the investigation of the response of *Phaseolus vulgaris* plants to soil application with biocompost in integration with chemical fertilizers applied to soil and plants. Four treatments (100% of the recommended NPK fertilizer dose (control), 75% NPK applied to soil + 25% foliar spray, 75% NPK applied to soil + 25% foliar spray + leguminous compost (C**_L_**), and 75% NPK applied to soil + 25% foliar spray + C**_L_** containing *Bacillus subtilis* (biocompost; C**_L_**B)) were applied in a randomized complete block design. The 75% NPK applied to soil + 25% foliar spray + C_L_B was the best treatment, which exceeded other treatments in improving soil fertility and plant performance. It noticeably improved soil physicochemical properties, including available nutrients, activities of various soil enzymes (cellulase, invertase, urease, and catalase), soil cation exchange capacity, organic carbon content, and pH, as well as plant growth and productivity, and plant physiobiochemistry, including nutrients and water contents, and various antioxidant activities. The results of the 2020 season significantly outperformed those of the 2019 season, indicating the positive effects of biofertilizers as a strategy to combine soil supplementation with NPK fertilizers and allocate a portion of NPK fertilizers for foliar spraying of plants in nutrient-deficient sandy soils.

## 1. Introduction

Sandy soils are characterized by low nutrient content and water holding capacity, which leads to the frequent application of nutrients and water to meet crop requirements and improve soil quality [1,2,3,4,5]. Virgin sandy soils, which were not used for cultivation before [6], are found in nearly all regions of Egypt, from a few hundred square kilometers to more than a hundred thousand kilometers, covering most of Egypt’s total area [7,8,9,10,11].

Soil can generally be enriched with nutrients by adding them in solid form or dissolved in water, and the plant can also be nourished directly with nutrients through foliar application. Historically, soil application is the most common fertilization practice, but it depends on several factors including soil and plant characteristics and the physiological state, as well as weather conditions [12]. Generally, the availability of most nutrients is limited in soils with higher calcium carbonate content [13], which are common throughout Egypt. However, foliar application improves the uptake and the efficiency of these nutrients [14,15]. Hence, to alleviate micro- and macronutrient deficiencies in sandy soils, foliar fertilization is increasingly adopted [16]. Foliar fertilization results in rapid nutrient absorption and avoidance of several environmental factors such as antagonism, leaching, and deposition of elements [17]. Hence, it can avoid some of the problems of challenges associated with soil application of nutrients in sandy soils. [17,18]. The foliar application does not substitute for soil application, but supplements it [19,20]. However, fertilization of major nutrients, especially NPK, is more effective via soil application, whereas secondary nutrients, e.g., calcium, magnesium, and sulfur, as well as micronutrients, e.g., zinc, iron, manganese, copper, molybdenum, and boron, proved to be more effective via foliar application [19].

Chemical fertilizers are essential for plant nutrition, but they can cause environmental pollution [21], particularly nitrogen, which leads to an increase of NO_3_^−^ ions in the soil [20]; phosphorous may also cause soil contamination with Cd^2+^, which is readily absorbed and translocated into different parts of the plant [22]. As one of the basic organic fertilizers, leguminous compost can be utilized to reduce the quantities of chemical fertilizers applied [23,24]. The use of leguminous compost has multiple benefits including sanitation, reduced mass and bulk, and a lower C/N ratio [21]. Besides, it has the potential to maintain the fertility of soils in agricultural systems [25,26]. Legume plants can contribute as much as 50–250 kg/ha of nitrogen [27,28], and their litter can be utilized as a highly valuable compost (organic fertilizer). In contrast, traditional organic materials, e.g., crop residues, animal manure, etc., cannot alone improve soil fertility, as they are usually not available in sufficient quantities and require intensive labor [29].

Continuous use of inorganic fertilizers negatively affects soil fertility as it eventually leads to reduced crop yields. Moreover, its cost is much higher compared with organic fertilizers, which impacts the overall profits of agricultural production. Hence, it is of prime importance to appropriately combine inorganic with organic fertilizers [23] to overcome the environmental impacts and maximize the overall income. The objective of the present study is to evaluate the effectiveness of chemical and organic fertilizers (leguminous biocompost) in improving the fertility of nutrient-deficient sandy soil and its reflection on the growth and yield of *Phaseolus vulgaris* using a foliar and/or soil addition. We hypothesized that the combined application of soil and foliar chemical fertilizers with a bio-organic fertilizer would improve the fertility of the investigated sandy soil along with the growth and production of *Phaseolus vulgaris* plants.

## 2. Materials and Methods

### 2.1. Experimental Preparation and Setup

Two field trials were conducted over two consecutive summer seasons (20 February 2019 and 25 February 2020). Each summer season was preceded by the fall season (the first of September 2018 and 2019), which was planted with Egyptian clover (*Trifolium alexandrinum*) crop mixed with the soil when plants were 10 weeks old. Then, the soil was irrigated and left until the main planting seasons (February 2019 and 2020).

Next to the Agriculture College (Fayoum University) trials station (29°17′06′′ N and 30°54′55′′ E), an area of sandy soil (not cultivated before) with an area of 600 m^2^ was employed for this study. This area was characterized by moderate weather throughout the experimental period during the two growing seasons. Average daily temperatures were 24.5 ± 2.5 °C, mean relative humidity was 64.6 ± 6.4%, and light/dark averaged throughout the day 12/12 h.

Bronco cultivar of *Phaseolus vulgaris* (L.) was obtained from the Horticultural Research Institute (Egyptian Center for Agricultural Research). A sterile solution of sodium hypochlorite at a concentration of 1% was prepared to be used to sterilize the surface of the seeds for 5 min, then the seeds were cleaned with distilled water. Then, the seeds were prepared for planting after air drying for 1 h.

Before sowing and after harvesting the crop in the 2019 and 2020 seasons, the top 0–20 cm of the soil was sampled (*n* = 5) and analyzed for soil chemical and physical properties, applying the methods detailed in Page et al. [30] and Klute and Dirksen [31].

The soil area specified for this study was divided into 40 experimental units of 10.5 m^2^ each, excluding the area of the main boundaries and irrigation canals (180 m^2^). Each unit consists of five rows of 3 m in length, and each row consists of 30 hills each planted with three seeds. After full emergence, seedlings were thinned to one hill^−1^, counting 150 plants 10.5 m^−2^. The recommended doses of NPK fertilizers (e.g., ammonium sulfate (20.5% N), Ca-superphosphate (15.5% P_2_O_5_), and K-sulfate (48% K_2_O)) were added to the soil at a rate of 380 kg N, 380 kg P_2_O_5_, and 190 kg K_2_O ha^−1^. During preparation, the soil was supplemented with a half-dose of N with a full dose of P and K. Thirty days after sowing (DAS), the soil was supplemented with another half-dose of N. These NPK amounts were applied at 100% of the recommended doses as a control treatment. The second treatment was 75% of the recommended NPK doses (0.30, 0.30, and 0.15 kg per 10.5 m^2^, respectively) applied to the soil, + 25% (0.10, 0.10, and 0.05 kg 10.5 m^−2^, respectively) applied as three foliar sprays (each spraying with a third of the 25%); 15, 30, and 45 days after sowing. Foliar sprays of NPK were performed with a hand atomizer, and the spray solution amount (to runoff) was sprayed, and Tween 20 (a few drops) was utilized as a surfactant. The third treatment was similar to the second treatment, in addition to leguminous compost that was added at 1.2 kg m^−2^. The fourth treatment was as the second treatment, in addition to leguminous compost (C**_L_**; 1.2 kg m^−2^) containing bacteria (*Bacillus subtilis*) as a biocompost fertilizer (C**_L_**B). The bacteria were prepared in a microbiological laboratory and added to the C**_L_** at approximately 0.6 × 10^9^ CFU g^−1^ along with humic acid at 0.01% and amino acids at 0.01% to form a C**_L_**B. The experimental treatments are summarized in Table 1.

All soil additions were spread manually on the soil surface after mixing with an appropriate amount of sand, then incorporated into the 20 cm upper layer. The treatments were secured for a randomized complete block design with ten unit (10.5 m^2^) replicates for each of the four treatments in both 2019 and 2020 seasons. All other practices recommended for standard cultivation for commercial *Phaseolus vulgaris* production were followed [32].

### 2.2. Compost Preparation

Twenty-five kg of green shoots of faba bean were incorporated into a similar weight containing different organic materials, including 0.5 kg and 0.5 kg of bulking agents and potassium humate (K–H), respectively, along with 12.0 kg and 12.0 kg of cattle manure and Egyptian clover plants, respectively, as N sources. For the compost mixture, green shoots of faba bean, bulking agents, potassium humate, cattle manure, and Egyptian clover plants contributed 50.0, 0.5, 0.5, 24.0, and 24.0%, respectively. After mixing well, these mixtures were composted in a pilot plant utilizing the turning-pile system in trapezoidal piles (dimensions of the base were 5.0 × 20.0 × 7.5 m in height, length, and width, respectively). From August to February, during the bio-oxidative phase, overturning was performed every two weeks for the piles, keeping the level of moisture in the range of 40–60% while monitoring the temperature. The obtained compost was analyzed, and the resulting data were as follows: pH = 7.5, EC = 2.1 dS m^−1^, content of organic matter = 19.6%, K = 152 g kg^−1^, P = 33 g kg^−1^, and N = 115 g kg^−1^.

### 2.3. Soil Sampling

Before sowing and after harvesting the *Phaseolus vulgaris* crop, three samples were randomly gathered from the 0–20 cm upper soil layer of three randomly selected experimental units and immediately transported to the laboratory and sieved with a 2 mm stainless-steel soil sampler.

### 2.4. Assessments of Soil CEC, Organic Matter, and Nutrient Contents

The procedures detailed in [30,31] were applied to evaluate each of cation exchange capacity (CEC), organic matter (%), CaCO_3_ (%), and nutrient contents (e.g., N, P, K, Fe, Mn, and Zn).

### 2.5. Soil Organic Carbon and Soil Enzyme Activity Determinations

Soil samples with a size of up to 2 mm were utilized to assess the organic C content and enzyme activities in the tested soil. The samples were randomly gathered from all experimental units of all tested treatments, including the comparison units. The wet oxidation–redox titration method of Carter and Gregorich [33] was applied to determine organic C content. Cellulases were determined as the system of an enzyme, by which cellulose was degraded and reducing sugars were released as the end product. Cellulase activity was expressed as mg sugars released g^−1^ dry soil, 37 °C, 24 h. Then, the procedure (anthrone colorimetric analysis) of Hope and Burns [34] was applied to determine the reducing sugars. The procedures detailed in both Schinner and von Mersi [35] and Miller [36] were applied to assay soil invertase (EC 3.2.1.26) activity (mg sugars released g^−1^ dry soil, 37 °C, 24 h) and the content of reducing sugars. The buffered procedure of Kandeler and Gerber [37] was applied to assay soil urease (EC 3.5.1.5) activity (μg NH_3_-N g^−1^ dry soil, 37 °C, 24 h). The back-titrating residual H_2_O_2_ with the KMnO_4_ procedure of Johnson and Temple [38] and Stepniewska et al. [39] had functioned to assay soil catalase (EC 1.11.1.6) activity (μmol H_2_O_2_ g^−1^ dry soil, 25 °C, 24 h).

Soil enzyme activities were evaluated using controls made by mixing the buffer solution with either the soil fractions or the substrate solution. The values were corrected by subtracting the combined absorbance values of the sample and substrate controls from those of the analytical samples.

### 2.6. Assessment of Growth and Yield Components

Fifty days after sowing, shoots of 10 plants were randomly gathered from each treatment (10 experimental units; one plant from each unit) to evaluate fresh weight (g), and after drying these shoots at 70 °C, dry weight was recorded after two or more constant weights of each shoot.

Sixty-two to 70 days after sowing (green pod marketing stage), 10 plants from each treatment (10 experimental units; one plant from each unit) were randomly assigned to assess the average pod weight (g) and total plant green pods (g). Eighty days after sowing (dry seed marketing stage), the remaining plants were applied for dry seeds’ weight (g per plant) after pods were picked to air dry for 3 d.

### 2.7. Leafy Nutrient Contents

Uniform leafy samples were collected from 10 plants randomly gathered from each treatment (10 experimental units; one plant from each unit) to be dried (at 70 °C for 72 h) and digested to assess leafy content of nutrients. The micro-Kjeldahl technique was applied to assess total N content (mg g^‒1^ DW). The content (mg g^‒1^ DW) of P was colorimetrically evaluated with the use of the reagent of stannous chloride–ammonium molybdate [40], following the extraction with NaHCO₃ [41]. The flame photometer (ELE Flame Photometer, Leighton Buzzard, UK) technique was utilized to assess the content (mg g^‒1^ DW) of K^+^. Besides this, the atomic absorption spectrophotometry apparatus was utilized to determine the contents (mg g^‒1^ DW) of Fe^2+^, Mn^2+^, and Zn^2+^ nutrients spectrophotometry [42].

### 2.8. Total Chlorophyll, Osmoprotectants, and Antioxidants Contents

The total chlorophyll content was spectrophotometrically (UV-160A, Spectrometer, Shimadzu, Kyoto, Japan) evaluated applying the method of [43], utilizing leaf discs (0.5 g). The leafy samples were homogenized with acetone solution (80%, *v*/*v*) and then the centrifugation process was practiced (3000× *g*, 20 min) for obtaining the supernatant to measure the absorbances on 470, 645, and 663 nm.

At the field level, standardized, uniform leaves were assigned to measure chlorophyll fluorescence at 360 μmol mol^−1^ CO_2_, 21 °C, and 600 μmol m^−2^ s^−1^ PPFD utilizing a potable pulse-modulated fluorometer (FMS-2, Hansatech, Norfolk, UK) according to the procedure of Li et al. [44]. The maximum quantum yield of photosystem II (PSII) (Fv/Fm) was computed using the Maxwell and Johnson [45] formulae. According to the equal absorption, photosynthesis PI_ABS_ was computed according to the formula of Clark et al. [46].

*Phaseolus vulgaris* leaf RWC was evaluated applying the procedure of Osman and Rady [47] with the use of leafy discs (*n* = 20) after exclusion of the midrib. The fresh, turgid, and dry mass were recorded for applying in the following formula:

RWC (%) = [(fresh mass − dry mass)/(turgid mass ‒ dry mass)] × 100

The Irigoyen et al. [48] method was used (using ethyl alcohol 96%, *v*/*v*) to extract and determine soluble sugar content (mg g^‒1^ DW) in the standardized, uniform leafy samples. The same leafy tissues were utilized to determine the content of free proline (μg g^‒1^ DW) following the Bates et al. [49] method. Besides this, the full procedures of Griffith [50] and Mukherjee and Choudhari [51] were applied for determining glutathione (nmol glutathione g^−1^ FW) and ascorbate (μmol g^−1^ FW) contents, respectively.

### 2.9. Assaying the Activities of Antioxidative Enzymes

Leafy samples (from all treatments, 0.5 g) were extracted applying the full Mukherjee and Choudhari [51] procedure. After centrifuging the homogenates (15,000× *g*, 10 min) the obtained supernatants were utilized to assay enzyme activities (guaiacol peroxidase (GPD), catalase (CAT), and superoxide dismutase (SOD)). The full procedures of Giannopolitis and Ries [52], Aebi [53], and Putter [54] were applied for assaying SOD (EC1.15.1.1), CAT (EC1.11.1.6), and GPD activities, respectively. For enzyme, nitro blue tetrazolium (NBT), H_2_O_2_, and guaiacol with H_2_O_2_ solutions were the substrates utilized, respectively. Besides this, the full method of [55] was applied for assessing the protein content.

### 2.10. Data Analysis

Analysis of data was performed with IBM^®^ SPSS^®^ (SPSS Inc., IBM Corporation, New York, NY, USA) Statistics Version 25 (2017) for Windows. To verify the normal distribution of data the Shapiro–Wilk test was used [56,57] for main effects and interactions. Due to the data having normal distribution (*p* ≤ 0.05), parametric statistical tests were applied. The two-way ANOVA was performed to highlight the effect of four fertilization treatments, two growing seasons, and the interaction between fertilization treatments and growing seasons. Two-way ANOVA, followed by a post hoc LSD test was used for multiple comparisons among means of fertilization treatments, growing seasons, and the interaction between fertilization treatments and growing seasons. All statistical tests were two-tailed, and a *p*-value less than or equal to 0.05 was considered statistically significant. A confidence interval was estimated at 95%.

## 3. Results

### 3.1. Soil Physical and Chemical Properties Response to S_CF75_ + F_CF25_ + C_L_B Supplementation for Two Seasons (2019 and 2020)

Data in Table 2 show the desired changes in soil characteristics during the two seasons (2019 and 2020) resulting from soil supplementation with S_CF75_ + F_CF25_ + C_L_B (chemical fertilizers applied to the soil at 75% of recommended NPK doses + chemical fertilizers applied as foliar sprays at 25% of recommended NPK doses + biocompost; leguminous compost (1.2 kg m^−2^) containing bacteria) as the best treatment. The soil composition of clay, silt, and sand was not changed in either of the 2019 and 2020 growing seasons with S_CF75_ + F_CF25_ + C_L_B application. Other characteristics (e.g., pH, EC, and CaCO_3_ content) were slightly affected.

### 3.2. Response of Soil Chemical Properties and Plant Performances to the Growing Season

**Figure 1:**
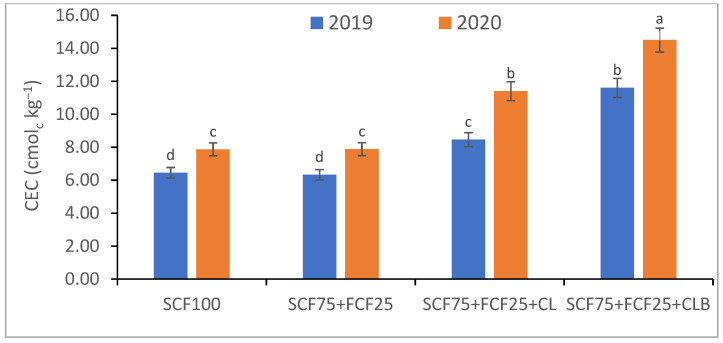
Response of cation exchange capacity to different fertilization strategies applied in two seasons (2019 and 2020) to sandy soil. Abbreviations of fertilization treatments are explained in Table 1. Bars with a different letter indicate significant difference between treatments at *p* ≤ 0.05.

**Figure 2:**
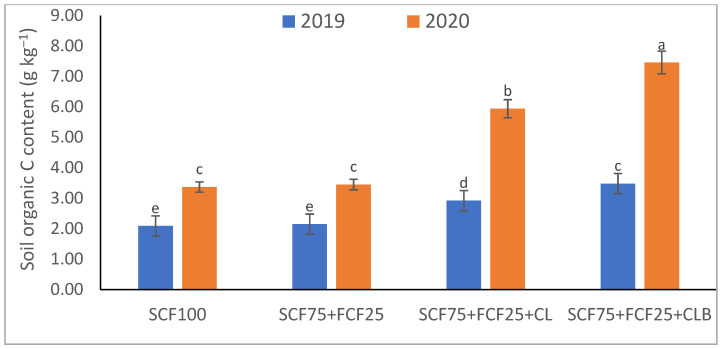
Response of soil organic C content to different fertilization strategies applied in two seasons (2019 and 2020) to sandy soil. Abbreviations of fertilization treatments are explained in Table 1. Bars with a different letter indicate significant difference between treatments at *p* ≤ 0.05.

**Figure 3:**
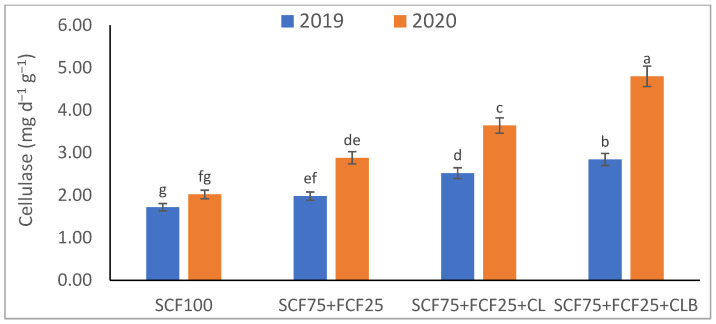
Response of soil enzyme activities (cellulase) to different fertilization strategies applied in two seasons (2019 and 2020) to sandy soil. Abbreviations of fertilization treatments are explained in Table 1. Bars with a different letter indicate significant difference between treatments at *p* ≤ 0.05.

**Figure 4:**
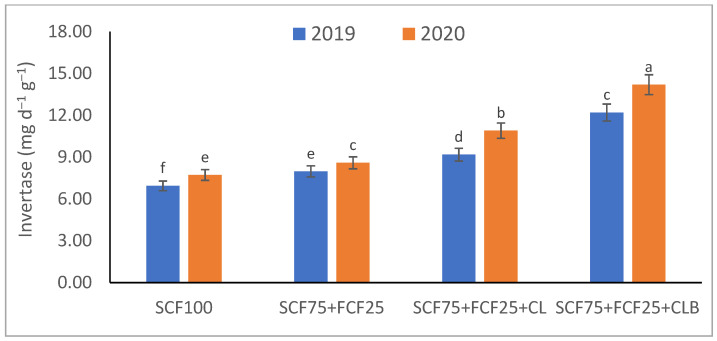
Response of soil enzyme activities (invertase) to different fertilization strategies applied in two seasons (2019 and 2020) to sandy soil. Abbreviations of fertilization treatments are explained in Table 1. Bars with a different letter indicate significant difference between treatments at *p* ≤ 0.05.

**Figure 5:**
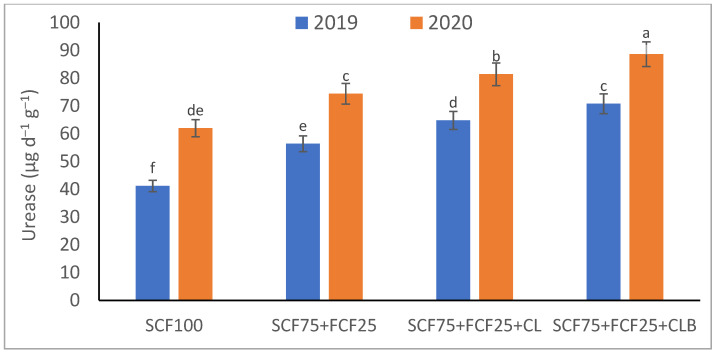
Response of soil enzyme activities (urease) to different fertilization strategies applied in two seasons (2019 and 2020) to sandy soil. Abbreviations of fertilization treatments are explained in Table 1. Bars with a different letter indicate significant difference between treatments at *p* ≤ 0.05.

**Figure 6:**
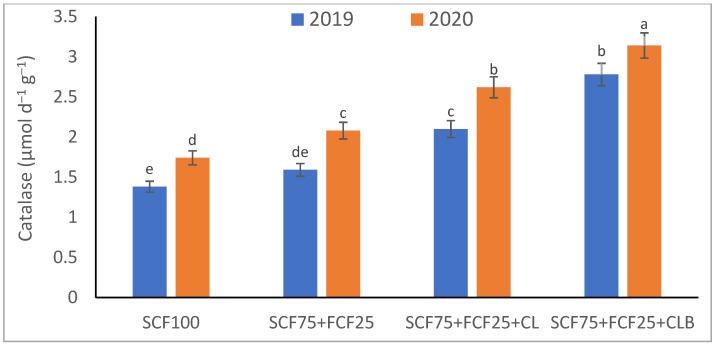
Response of soil enzyme activities (catalase) to different fertilization strategies applied in two seasons (2019 and 2020) to sandy soil. Abbreviations of fertilization treatments are explained in Table 1. Bars with a different letter indicate significant difference between treatments at *p* ≤ 0.05.

**Figure 7:**
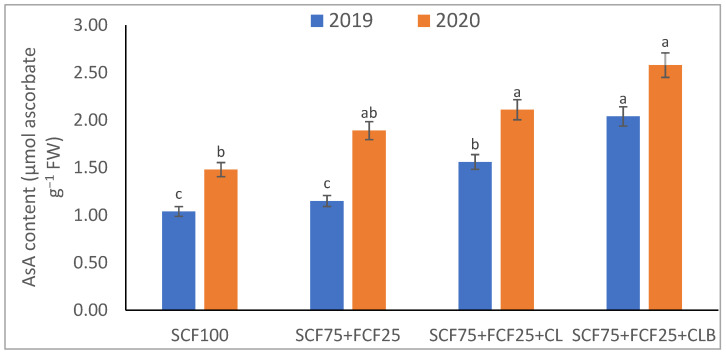
Response of leaf nonenzymatic antioxidants contents (ascorbic acid—AsA) of *Phaseolus vulgaris* plants to different fertilization strategies applied in two seasons (2019 and 2020) to sandy soil. Abbreviations of fertilization treatments are explained in Table 1. Bars with a different letter indicate significant difference between treatments at *p* ≤ 0.05.

**Figure 8:**
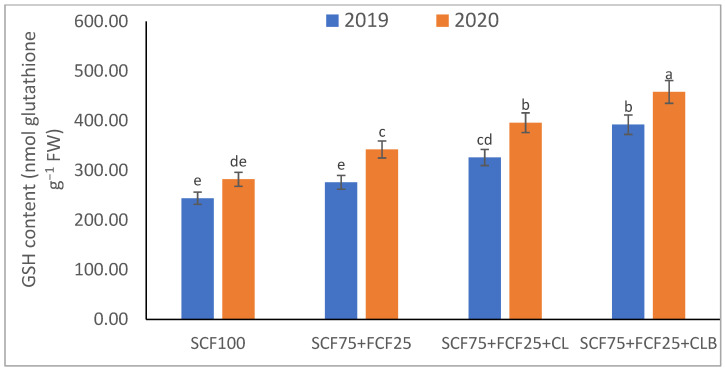
Response of leaf nonenzymatic antioxidants contents (glutathione—GSH) of *Phaseolus vulgaris* plants to different fertilization strategies applied in two seasons (2019 and 2020) to sandy soil. Abbreviations of fertilization treatments are explained in Table 1. Bars with a different letter indicate significant difference between treatments at *p* ≤ 0.05.

**Figure 9:**
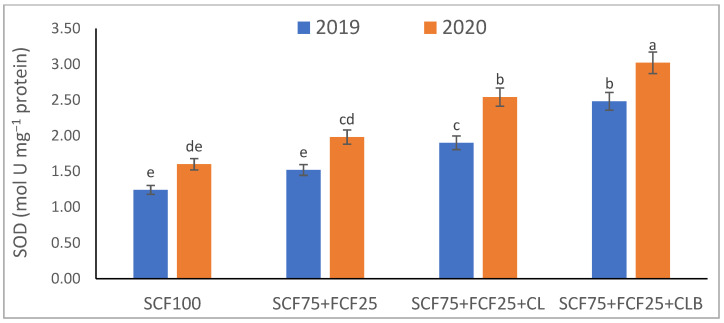
Response of leaf enzyme activity (superoxide dismutase—SOD) of *Phaseolus vulgaris* plants to different fertilization strategies applied in two seasons (2019 and 2020) to sandy soil. Abbreviations of fertilization treatments are explained in Table 1. Bars with a different letter indicate significant difference between treatments at *p* ≤ 0.05.

**Figure 10:**
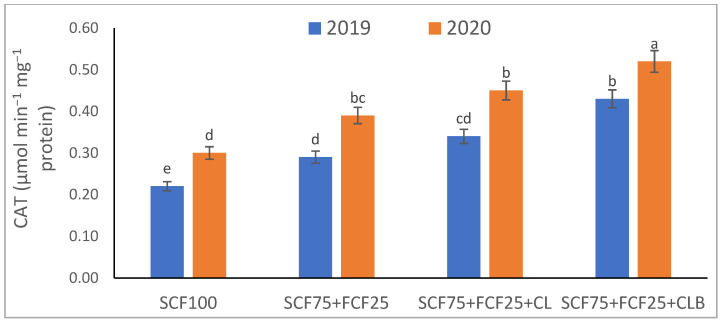
Response of leaf enzyme activity (catalase—CAT) of *Phaseolus vulgaris* plants to different fertilization strategies applied in two seasons (2019 and 2020) to sandy soil. Abbreviations of fertilization treatments are explained in Table 1. Bars with a different letter indicate significant difference between treatments at *p* ≤ 0.05.

**Figure 11:**
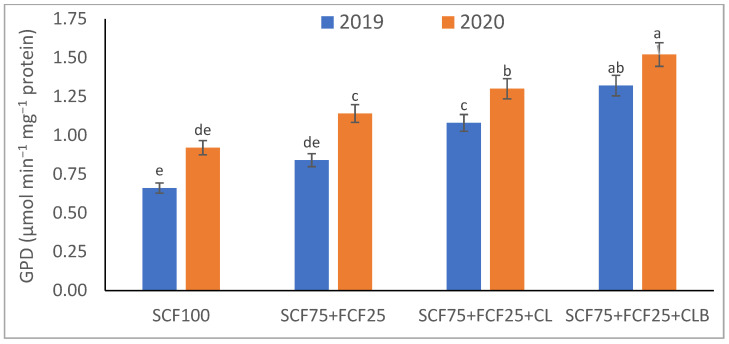
Response of leaf enzyme activity (guaiacol peroxidase—GPD) of *Phaseolus vulgaris* plants to different fertilization strategies applied in two seasons (2019 and 2020) to sandy soil. Abbreviations of fertilization treatments are explained in Table 1. Bars with a different letter indicate significant difference between treatments at *p* ≤ 0.05.

**Figure 12:**
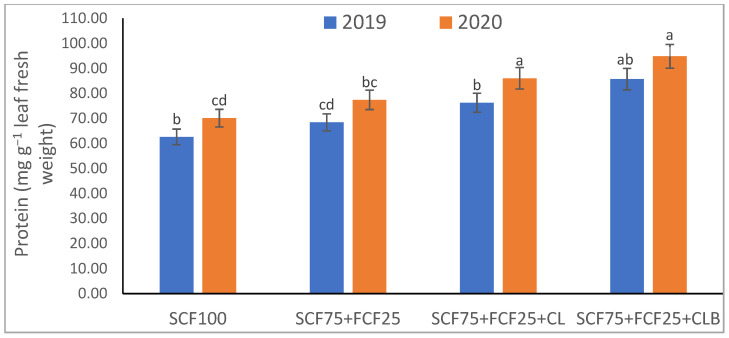
Response of leaf enzyme activity (protein) of *Phaseolus vulgaris* plants to different fertilization strategies applied in two seasons (2019 and 2020) to sandy soil. Abbreviations of fertilization treatments are explained in Table 1. Bars with a different letter indicate significant difference between treatments at *p* ≤ 0.05.

The growing season as the main factor in this study affected the chemical properties of the tested soil, soil and plant contents of essential nutrients, activities of various enzymes, plant growth and productivity, and plant physiobiochemistry. The 2020 growing season significantly outperformed the 2019 growing season in terms of all the examined parameters of the soil and *Phaseolus vulgaris* plant, which are presented in Table 3, Table 4, Table 5 and Table 6 and Figure 1, Figure 2, Figure 3, Figure 4, Figure 5, Figure 6, Figure 7, Figure 8, Figure 9, Figure 10, Figure 11 and Figure 12.

In the 2020 growing season, there were significant (*p* ≤ 0.05) increases in available N at 18.5%, K at 21.3%, Fe at 31.9%, Mn at 45.7%, and Zn at 42.3% compared to those obtained in the 2019 growing season (Table 3). The data in Figure 1, Figure 2, Figure 3, Figure 4, Figure 5 and Figure 6 display that the cation exchange capacity (CEC), organic C content, cellulose, invertase, urease, and catalase activities of the tested soil were significantly increased in the 2020 growing season compared to those achieved in the 2019 season. As shown in Table 4, shoot fresh and dry weights, and green pods and dry seed weights of *Phaseolus vulgaris* plants were significantly (*p* ≤ 0.05) increased by 20.8, 15.6, 14.5, and 28.4%, respectively, in the 2020 season compared to those gained in the 2019 season. The plant nutrient contents represented the same behavior of the soil nutrient contents, since N, P, K, Fe, Mn, and Zn contents were significantly (*p* ≤ 0.05) increased by 19.9, 25.4, 15.8, 17.5, 23.9, and 19.8% in the 2020 growing season compared to those collected in the 2019 season (Table 5). The data in Table 6 and Figure 7, Figure 8, Figure 9, Figure 10, Figure 11 and Figure 12 revealed that the physiobiochemical attributes (e.g., total chlorophyll content, Fv/Fm, PSII performance index, leaf relative water content, proline content, total soluble sugar content, ascorbate content, glutathione content, protein content, and leaf enzymatic activity of guaiacol peroxidase, catalase, and superoxide dismutase) of *Phaseolus vulgaris* plants were significantly elevated in the 2020 season compared to those determined in the 2019 season. The total chlorophyll content, Fv/Fm, PSII performance index, leaf relative water content, proline content, total soluble sugar contents were increased by 21.6, 7.8, 16.1, 13.5, 15.6, and 24.4%, respectively. Besides this, the contents of ascorbate, glutathione, and protein, as well as the activities of guaiacol peroxidase, catalase, and superoxide dismutase, were increased by 39.3, 19.4, 27.9, 31.3, 24.5, and 12.2, respectively.

### 3.3. Response of Soil Chemical Properties and Plant Performances to Different Fertilization Strategies

Some fertilization strategies (sub-main factor) were applied to the nutrient-deficient virgin sandy soil tested in this study, and they positively influenced the soil chemical properties, soil and plant contents of essential nutrients and activities of various enzymes, plant growth and productivity, and plant physiobiochemistry (Table 3, Table 4, Table 5 and Table 6 and Figure 1, Figure 2, Figure 3, Figure 4, Figure 5, Figure 6, Figure 7, Figure 8, Figure 9, Figure 10, Figure 11 and Figure 12).

All blended fertilization strategies used in this study significantly increased the chemical properties of the tested soil, soil and plant contents of essential nutrients and activities of various enzymes, plant growth and productivity, and the plant physiobiochemistry, excluding some exceptions recorded by S_CF75_ + F_CF25_ treatment compared to control (S_CF100_ = chemical fertilizers applied to the soil at full recommended NPK doses). Among all fertilization strategies, the S_CF75_ + F_CF25_ + C_L_B was the best treatment. It conferred (*p* ≤ 0.05) significant increases in soil content of available N, Mn, and Zn by 56.7, 61.3, and 50.9%, respectively (Table 3), and soil cellulase and urease activities by 80.1 and 54.5%, respectively (Figure 3 and Figure 5), compared to control. Besides this, it awarded highly significant (*p* ≤ 0.01) increases in soil content of available P, K, and Fe by 84.7, 58.9, and 82.6%, respectively (Table 3), and soil CEC, organic C content, and invertase and catalase activities by 83.0, 100.4, 104.3, and 89.7%, respectively (Figure 4 and Figure 6), compared to control. For *Phaseolus vulgaris* plant, the best treatment (S_CF75_ + F_CF25_ + C_L_B) also conferred highly significant (*p* ≤ 0.01) increases in shoot fresh weight and dry seed weight by 70.6 and 109.6%, respectively (Table 4), contents of P, Mn, Zn, total chlorophylls, proline, soluble sugars, and ascorbate by 98.6, 83.3, 76.0, 103.8, 50.7, 85.9, and 83.3%, respectively (Table 5 and Table 6; Figure 7), and activities of superoxide dismutase, catalase, and guaiacol peroxidase by 93.7, 84.6, and 79.7%, respectively (Figure 9, Figure 10 and Figure 11), compared to control. It contributed significant (*p* ≤ 0.05) increases in shoot dry weight and green pods weight by 51.3 and 56.9%, respectively (Table 4), contents of N, K, Fe, glutathione, and protein by 73.4, 68.0, 71.0, 61.6, and 36.0%, respectively (Table 5; Figure 8 and Figure 12), and in Fv/Fm, performance index, and relative water content by 25.4, 60.9, and 45.1%, respectively (Table 6), compared to control (S_CF100_).

### 3.4. Response of Soil Chemical Properties and Plant Performances to the Interaction between Growing Season and Different Fertilization Strategies

The interaction between growing season and fertilization strategies and their influences on the chemical properties of the tested soil, soil and plant contents of essential nutrients and activities of various enzymes, plant growth and productivity, and plant physiobiochemistry under nutrient-deficient virgin sandy soil conditions are shown in Table 3, Table 4, Table 5 and Table 6 and Figure 1, Figure 2, Figure 3, Figure 4, Figure 5, Figure 6, Figure 7, Figure 8, Figure 9, Figure 10, Figure 11 and Figure 12.

Among all interactions, the application of 2019 × S_CF100_ (control) or 2019 × S_CF75_ + F_CF25_ collected the lowest findings of all determined soil and plant parameters. However, 2020 × S_CF75_ + F_CF25_ + C_L_B was the best interaction treatment, which conferred significant (*p* ≤ 0.05) or highly significant (*p* ≤ 0.01) increases in all tested soil and plant parameters. It conferred (*p* ≤ 0.05) significant increases in soil content of available N, Mn, and Zn by 82.0, 149.1, and 120.7%, respectively (Table 3), and soil cellulase and urease activities by 104.6 and 115.0%, respectively (Figure 3 and Figure 5), compared to control. Besides, it contributed highly significant (*p* ≤ 0.01) increases in soil content of available P, K, and Fe by 131.7, 91.0, and 131.5%, respectively (Table 3), and soil CEC, organic C content, and invertase and catalase activities by 124.8, 256.9, 179.1, and 127.5%, respectively (Figure 1, Figure 2, Figure 3, Figure 4, Figure 5 and Figure 6), compared to control. For *Phaseolus vulgaris* plant, the best interaction treatment (2020 × S_CF75_ + F_CF25_ + C_L_B) also conferred highly significant (*p* ≤ 0.01) increases in shoot fresh weight and dry seed weight by 102.1 and 165.2%, respectively (Table 4), contents of P, Mn, Zn, total chlorophylls, proline, soluble sugars, and ascorbate by 159.0, 121.9, 109.0, 155.4, 74.4, 132.3, and 124.3%, respectively (Table 5 and Table 6; Figure 7), and activities of superoxide dismutase, catalase, and guaiacol peroxidase by 143.5, 136.4, and 130.3%, respectively (Figure 9, Figure 10 and Figure 11), compared to control. It contributed significant (*p* ≤ 0.05) increases in shoot dry weight and green pods weight by 75.4 and 82.7%, respectively (Table 4), contents of N, K, Fe, glutathione, and protein by 103.9, 94.4, 100.0, 87.7, and 51.4%, respectively (Table 5; Figure 8 and Figure 12), and in Fv/Fm, performance index, and relative water content by 35.3, 90.4, and 65.6%, respectively (Table 6), compared to control (S_CF100_).

## 4. Discussion

Climate change, as one of the major challenges facing the world at present, has negatively affected agricultural lands. Furthermore, future changes in climatic events are expected to worsen [58], particularly in arid and semi-arid regions [59,60,61,62,63]. As a consequence, soils have lost much of their fertility, threatening the food security of the world [58,64,65,66,67,68,69,70]. Accordingly, to maintain sustainable agricultural development, virgin (unused) lands that can be cultivated with more organic and less chemical fertilization, are being, or should be, used to minimize pollutants to produce clean agricultural products free of pollution [71].

Sandy soils suffer from high permeability coupled with poor water retention, which results in the loss of many important nutrients due to poor ability to retain many important nutrients, besides, the competition of cations, and consequently the rapid leaching [1,72,73,74]. Besides, if such soil was virgin, its suffering would have been greater. Therefore, it is necessary to improve the weaknesses of this soil by nourishing it with organic fertilizers, especially biocomposts, because they enhance microbial decomposition leading to humification of organic matter [75] in favor of the soil and plants growing on it.

The virgin sandy soil used in this study had very low water and nutrient holding capacity and a poor cation exchange capacity (CEC), making it impossible to rely on adding nitrogen, phosphorus, and potassium (NPK) fertilizers only, otherwise, these fertilizers are rapidly lost. Therefore, to minimize fertilizer nutrient loss, it is appropriate to add a portion of the recommended NPK fertilizer (about 25%) to the plant as a foliar spray in combination with adding another portion (about 75%) to the soil with the addition of an appropriate amount of organic (S_CF75_ + F_CF25_ + C**_L_**) or bio-organic fertilizer (S_CF75_ + F_CF25_ + C_L_B) as a required strategy for the sustainability and productivity of plants growing on the tested soil. From this point of view, the S_CF75_ + F_CF25_ treatment went beyond the control treatment and significantly increased soil physicochemical properties (Table 2 and Table 3; Figure 1, Figure 2, Figure 3, Figure 4, Figure 5 and Figure 6) and *Phaseolus vulgaris* plant growth, productivity, and physiobiochemical attributes (Table 4, Table 5 and Table 6; Figure 7, Figure 8, Figure 9, Figure 10, Figure 11 and Figure 12). Enrichment of the S_CF75_ + F_CF25_ treatment with leguminous compost (C**_L_**) at 1.2 kg per m^2^ (S_CF75_ + F_CF25_ + C**_L_**) increased the treatment efficiency, as it exceeded the S_CF75_ + F_CF25_ treatment, resulting in a significant increase in the tested soil and plant attributes (Table 3, Table 4, Table 5 and Table 6; Figure 1, Figure 2, Figure 3, Figure 4, Figure 5, Figure 6, Figure 7, Figure 8, Figure 9, Figure 10, Figure 11 and Figure 12). Moreover, enrichment of the C**_L_** in the S_CF75_ + F_CF25_ + C**_L_** treatment with bacteria (*Bacillus subtilis*) further increased the treatment efficiency, as the S_CF75_ + F_CF25_ + C_L_B treatment greatly exceeded the S_CF75_ + F_CF25_ + C**_L_** treatment, resulting in a further significant improvement in soil and plant attributes (Table 2, Table 3, Table 4, Table 5 and Table 6; Figure 1, Figure 2, Figure 3, Figure 4, Figure 5, Figure 6, Figure 7, Figure 8, Figure 9, Figure 10, Figure 11 and Figure 12).

Therefore, from our findings of the tested virgin sandy soil and *Phaseolus vulgaris* plant, the S_CF75_ + F_CF25_ + C_L_B treatment was the best, with the best improvement results for the soil texture, pH, EC_e_, and CaCO_3_ content in both the 2019 and 2020 seasons (Table 2, Table 3, Table 4, Table 5 and Table 6; Figure 1, Figure 2, Figure 3, Figure 4, Figure 5, Figure 6, Figure 7, Figure 8, Figure 9, Figure 10, Figure 11 and Figure 12). These improvements were attributed to the leguminous biocompost (C**_L_**B), which provided soil characteristics, in addition to the NPK nutrients that the plant acquired through two pathways: roots and leaves. In addition, compared to all treatments, including the control, the S_CF75_ + F_CF25_ + C_L_B treatment significantly increased CEC, soil organic carbon (C) content, soil nutrient (N, P, K, Fe, Mn, and Zn) contents, and soil enzymes (e.g., cellulase, invertase, urease, and catalase) activities (Table 3 and Figure 1, Figure 2, Figure 3, Figure 4, Figure 5 and Figure 6).

Soil pH was decreased with soil supplementation using C**_L_**B, which could be attributed to the increase in organic C content and soil enzyme activities (Figure 1, Figure 2, Figure 3, Figure 4, Figure 5 and Figure 6), which led to the decomposition of the added organic matter (in C**_L_**B). Besides, organic acids and phytohormones (e.g., indole acetic acid and cytokinins) resulting from the activity of bacteria added to the compost, leading to an increase in biological activity [33,76,77,78]. As obtained in this study (Table 2), a decrease in pH is reported with the combined use of biocompost and inorganic fertilizer [79]. This positive result is attributed to the production of organic acids due to biocompost decomposition followed by an increase in the salt content of the soil due to mineralization, which increases the EC of the soil. Sinha et al. [79] also reported that soil pH decreased while EC increased, due to biocompost application, which also had a significant influence on soil organic C content and available nutrients (e.g., N, P, K, and S).

Furthermore, supplementing the soil with C**_L_**B enhanced the microbial decomposition that leads to an increase in soil organic matter and thus CEC (Figure 1, Figure 2, Figure 3, Figure 4, Figure 5 and Figure 6). Buragohain et al. [75] found that the biocompost generates a large number of different bacteria that cause an increase in soil organic matter, which leads to improved crop growth. This subsequently results in increased exudations of plant roots and return of post-harvest residues, thus contributing to the organic content of the soil. They also documented that the observed high contribution of biocompost to soil organic C indicates more rebellious forms of soil organic C, thus resulting in enhanced soil C stabilization. Soil organic C elevation is also observed following the application of biofertilizers (*Bacillus megatherium* and *Bacillus mucilaginous*), as per Wu et al. [80]. Increase of CEC after application of compost [21], and in particular biocompost, is an indication of a high accumulation of soil organic C pool along with low inorganic P and N. The decreased inorganic P and N following the application of biocompost contribute to building a soil environment where a desirable microbial composition is created to stabilize the soil organic carbon pool [75]. They also reported that the quality of organic matter and fertility of the soil is improved due to the increased humic/fulvic acid ratio due to the increased rate of humification, which helps to raise the stability of organic C as a result of biocompost supplementation to the soil [81].

The buildup of available nutrients in nutrient-deficient soils, such as the soil tested in this study, can be attributed to the increased microbial proliferation due to the addition of organic manures, especially biocompost, which helps in mineralization as well as solubilization of native nutrients by complexation of nutrients by humic and fulvic acid contained in biocompost [79]. The results of this study indicated that the application of NPK fertilizers only was not effective in maintaining soil fertility (Table 2 and Table 3; Figure 1, Figure 2, Figure 3, Figure 4, Figure 5 and Figure 6). The nutrients available in the soil and soil organic C were preserved in the treatment containing biocompost. It has been reported by Sinha et al. [79] that soil fertility-contributing bulk density and pore spaces are improved effectively due to the accumulation of organic C content of the biocompost-treated soil. The beneficial influences of biocompost in improving the soil’s physical and chemical properties may be attributed to the improvement in the organic matter status of the soil treated with biocompost, which leads to the buildup of soil fertility for sustainable production of *Phaseolus vulgaris*. Sinha et al. [79] also explained that the use of biocompost greatly increases the soil microbial population, which utilizes the accumulated organic C as a source of energy, nutrients, and nourishment, which leads to the spread of microorganisms in the soil. This improved microbial community and activity due to the accumulation of organic matter by the application of biocompost help maintain soil fertility and productivity due to the faster decomposition rate and smooth mineralization of organic materials [79].

As shown in the data of this study, the combined use of biocompost and inorganic fertilizers (applied to both soil and plant leaves) effectively increased the uptake of nutrients, which reflected positively on the higher yield (Table 4 and Table 5). Managing nutrients through organic and inorganic sources leads to more nutrient uptake. As of the effectively increased parameters due to the combined use of C**_L_**B and inorganic fertilizers, the soil organic C content and availability of nutrients (e.g., N, P, K, Fe, Mn, and Zn) in the tested virgin sandy soil may be attributed to the increased content of organic C and nutrients in the C**_L_**B and the beneficial advantages of inorganic nutrients. These nutrients are released from the compost into the soil through bacterial decomposition [82] present in C**_L_**B, which is activated by the added compost. Manirakiza and Şeker [77] reported increased contents of soil nutrients and organic C which could be attributed to the compost’s richness in organic C and various nutrients. The release of nutrients from compost into the soil via the mineralization process can elucidate this result [77,83].

Compared to all other treatments, including the control, the S_CF75_ + F_CF25_ + C_L_B treatment significantly increased soil enzymes (e.g., cellulase, invertase, urease, and catalase) activities, especially after adding C**_L_**B, indicating a potentially greater source of beneficial microbes. The increased activity of soil enzymes contributed to the release of more nutrients for microbial use [75]. Besides, the bacteria present in the C**_L_**B have desirable influences on increasing and stabilizing bacterial populations [84], which contributed along with the increased soil enzyme activities to repair limitations of the soil tested.

Macci et al. [85] and Buragohain et al. [75] demonstrated that compost and biocompost fertilizers increase soil quality due to enhancements of soil properties related to physicochemistry and biochemistry. This positive finding is confirmed by the results obtained for the tested virgin sandy soil, which reflected positively in the growth, productivity, and physiobiochemical indices of *Phaseolus vulgaris* plants (Table 4, Table 5 and Table 6; Figure 7, Figure 8, Figure 9, Figure 10, Figure 11 and Figure 12). The increased availability of nutrients to the plants as a result of the improved physicochemical properties of the tested soil under supplementation of biocompost contributed to the increase in growth and physiobiochemical indices, and thus the productivity of *P. vulgaris* plants. As reported in [86], compost enriched with many bacterial strains (e.g., *Bacillus*, *Rhizobium*, *Azotobacter*, *Azospirillum*, *Bradyrhizobium*, *Acetobacter*, and *Pseudomonas*) has been reported to improve yields of different crops in some defective soils. Furthermore, it has been explained by Sinha et al. [79] that the immediate and rapid supply of nutrients through an inorganic source for plant growth and a steady supply of nutrients by organics, especially biocompost, throughout the growth period leads to an increase in plant yield due to integrated use of organic and inorganic nutrients. The biocompost releases nutrients after decomposition and mineralization that will increase the availability of nutrients at a later stage and improve physical, chemical, and biological properties of soil, resulting in improved soil fertility and absorption of nutrients by the plant. The integrated use of organic nutrient sources with inorganic fertilizers has been shown to increase the potential of organic fertilizers [79].

The growth and yield trend after two consecutive trial years of *P. vulgaris* plants showed that reducing 25% of the recommended NPK fertilizer dose of soil addition for use in foliar spraying along with the application of biocompost resulted in a progressive increase in the growth and yield of *P. vulgaris* plants (Table 4). Thus, the S_CF75_ + F_CF25_ + C_L_B treatment represented the highest sustainability for a period of two seasons (2019 and 2020). Allocating 25% of the NPK fertilizers to foliar spray conferred the opportunity to reduce the inorganic minerals added to the soil along with the use of C**_L_**B to conserve the soil while increasing its fertility (Table 2 and Table 3; Figure 1, Figure 2, Figure 3, Figure 4, Figure 5 and Figure 6). Besides, it increased the efficiency of the growing *P. vulgaris* plants in sandy soil to obtain all their nutritional needs, especially in the presence of C**_L_**B that facilitates nutrient uptake. Foliar spraying with NPK nutrients has become a concern of scientists for its dynamic application to increase plant growth and yield [17,87] (Table 2, Table 3, Table 4, Table 5 and Table 6; Figure 1, Figure 2, Figure 3, Figure 4, Figure 5, Figure 6, Figure 7, Figure 8, Figure 9, Figure 10, Figure 11 and Figure 12).

As explained in [88], the increase in plant growth and productivity can be attributed to the positive influences of biocompost and its content of microorganisms in raising root surfaces, root distribution, water-use efficiency, and photosynthetic activity. These positive results directly affect the physiological processes and carbohydrate metabolism due to the high levels of nutrients and organic matter in the applied biocompost. Similarly, our data attributed the increases in growth and productivity of *P. vulgaris* plants to the application of C**_L_**B, which contributed to improved phytonutrient contents (Table 3), photosynthetic efficiency (chlorophyll content, chlorophyll fluorescence, and PSII performance index), and relative cellular water content (Table 4), as well as the plant’s antioxidant defense system; osmoprotectants (proline and sugars) and both enzymatic and nonenzymatic antioxidants (Table 6 and Figure 7, Figure 8, Figure 9, Figure 10, Figure 11 and Figure 12).

The physicochemical properties and fertility of the virgin sandy soil, including the soil’s organic C content and organic acids, which were greatly improved by applying C**_L_**B (Table 2 and Table 3; Figure 1, Figure 2, Figure 3, Figure 4, Figure 5 and Figure 6), led to an increase in water retention in the soil [89], allowing *P. vulgaris* plants to absorb more water and nutrients, leading to increased relative cellular water content and different nutrient contents (Table 4, Table 5 and Table 6). Relative cellular water content indicates the water status in plants, reflecting the activity of plant metabolic processes, and is utilized as an indicator to distinguish between legumes with contrasting variations in drought tolerance [90]. The increased plant water and nutrient contents through the application of S_CF75_ + F_CF25_ + C_L_B played crucial roles in improving the efficiency of photosynthesis and the antioxidant defense system (Table 4 and Figure 7, Figure 8, Figure 9, Figure 10, Figure 11 and Figure 12), which need water as a medium for their reactions to take place [22,91]. The S_CF75_ + F_CF25_ + C_L_B, including C**_L_**B, notably improved *P. vulgaris* plant water content due to the improved water uptake by roots and as a result of the accumulation of elemental K^+^, free proline, and soluble sugars as osmoprotectant compounds [47,73]. Furthermore, the accumulation of ascorbate and glutathione with the S_CF75_ + F_CF25_ + C_L_B treatment contributed to the improved tissue water status and membrane integrity by reducing the activity of the reactive oxygen species (ROS) [47,92,93]. It has been explained that the accumulation of K^+^, free proline, and soluble sugars as osmolytes contributes to osmotic adjustment, which helps to maintain the cell turgor and stabilize the membranes by activation of antioxidant plant defense system [73,94,95,96,97,98,99]. This is also evident from improvement in antioxidant enzymes activities in this study (Figure 7, Figure 8, Figure 9, Figure 10, Figure 11 and Figure 12), such as superoxide dismutase (SOD), catalase (CAT), and guaiacol peroxidase (GPOD), including leaf content of protein for the S_CF75_ + F_CF25_ + C_L_B treatment in *P. vulgaris* plants grown under the tested conditions. Enhanced activities of assayed antioxidant enzymes with the S_CF75_ + F_CF25_ + C_L_B treatment have been also associated with improved photosynthetic activity and carbohydrates supply to growing sink [73], which contributed towards increased growth and yield of *P. vulgaris* plants. This increase in *P. vulgaris* plant performance with the S_CF75_ + F_CF25_ + C_L_B treatment is also associated with its influences on different physiological mechanisms and enzymes such as starch metabolism and glucose transport during photosynthesis and accumulation period [100]. All these findings were reflected, positively, in *P. vulgaris* plant growth and productivity (Table 4).

Soil application with C**_L_**B (compost + bacteria) can integrate conventional NPK fertilizers that should be applied to both soil and plant (foliar spray) in the cultivation of *P. vulgaris* and other crops using the defective sandy soils to improve plants’ efficiency for good production and minimize contamination of farmland and agricultural products achieving agricultural sustainability.

All results of the 2020 season notably exceeded those of the 2019 season in terms of all the attributes examined for soil physicochemical properties and *P. vulgaris* plant growth, yield, and physiobiochemistry (Table 2, Table 3, Table 4, Table 5 and Table 6; Figure 1, Figure 2, Figure 3, Figure 4, Figure 5, Figure 6, Figure 7, Figure 8, Figure 9, Figure 10, Figure 11 and Figure 12). This may be attributed to the residual effects of the first year’s treatments, which provided the opportunity to liberate nutrients in excess amounts from the soil due to the elevated bacterial decomposition of the biocompost applied in the preceding season (2019), as well as the increased solubility of nutrients that contained in the biocompost.

## 5. Conclusions

The positive influences of leguminous biocompost in integration with reducing 25% of the recommended NPK fertilizers on soil fertility and physicochemical properties, as well as *P. vulgaris* plant growth, productivity, and physiobiochemical attributes, have been elucidated in a virgin sandy soil. Soil application with leguminous biocompost at a rate of 1.2 kg per m^2^ + 75% of the recommended NPK fertilizer doses in integration with 25% of the recommended NPK fertilizer doses as a foliar spray for *P. vulgaris* plants resulted in significant improvements in soil fertility, physicochemical properties and plant physiobiochemical attributes, which were positively reflected in the plant growth and yield. These positive soil and plant results indicate the positive influences of biofertilizers as a supplementary fertilizer strategy for integrating soil application with NPK fertilizers, allocating a portion of mineral fertilizers for foliar spraying for sustainable agriculture using virgin sandy soils that can be used for the expansion of crop cultivation.

## Figures and Tables

**Table 1:** The description of the experimental fertilization treatments for soil and plant.

Treatment	Description
1. S_CF100_	100% of the recommended NPK doses were applied to the soil as a control.
2. S_CF75_ + F_CF25_	75% of the recommended NPK doses were applied to the soil, +25% of the recommended NPK doses were applied as three foliar sprays (each spraying with the third of the 25%).
3. S_CF75_ + F_CF25_ + C_L_	75% of the recommended NPK doses were applied to the soil, +25% of the recommended NPK doses were applied as three foliar sprays (each spraying with the third of the 25%) + 1.2 kg leguminous compost m^−2^.
4. S_CF75_ + F_CF25_ + C_L_B	75% of the recommended NPK doses were applied to the soil, +25% of the recommended NPK doses were applied as three foliar sprays (each spraying with the third of the 25%) + 1.2 kg biocompost (leguminous compost containing bacteria; *Bacillus subtilis*) m^−2^.

**Table 2:** The physicochemical properties of the tested soil before sowing and following and after harvest.

Parameters	Properties			
	2019		2020	
	Before Sowing	After Harvest	Before Sowing	After Harvest
Clay	8.2 ± 0.6 *	8.2 ± 0.5	8.2 ± 0.7	8.3 ± 0.8
Silt	10.7 ± 0.8	10.8 ± 0.8	10.8 ± 0.7	10.9 ± 1.0
Sand	81.1 ± 7.2	81.0 ± 6.9	81.0 ± 6.7	80.8 ± 6.2
Soil texture	Loamy Sand	Loamy Sand	Loamy Sand	Loamy Sand
pH	8.35 ± 0.51	8.12 ± 0.48	8.02 ± 0.42	7.82 ± 0.38
EC (dS m^−1^)	3.20 ± 0.24	3.24 ± 0.24	3.31 ± 0.26	3.44 ± 0.25
CaCO_3_ (%)	8.62 ± 0.54	8.52 ± 0.55	8.44 ± 0.50	8.10 ± 0.48
Available nutrients (mg kg^−1^ soil) before sowing				
N	12.7 ± 0.2		27.8 ± 0.5	
P	7.6 ± 0.1		15.3 ± 0.3	
K	16.4 ± 0.3		40.1 ± 0.8	

* Values ± SE, dS m^−1^ = deciSiemens per meter, and S_CF75_ + F_CF25_ + C_L_B = chemical fertilizers applied to the soil at 75% of recommended NPK doses + chemical fertilizers applied as foliar sprays at 25% of recommended NPK doses + biocompost; leguminous compost (1.2 kg m^−^^2^) containing bacteria.

**Table 3:** Response of soil nutrient contents to growing season (2019 and 2020) and different fertilization strategies applied to virgin sandy soil.

**Source of Variation**	**Available Macronutrient Contents (mg kg^−1^ Soil)**		
	**Available N**	**Available P**	**Available K**
Season (S)	*	ns	*
2019	30.3 ± 0.2 ^b^	17.5 ± 0.2 ^a^	37.5 ± 0.3 ^b^
2020	35.9 ± 0.3 ^a^	17.5 ± 0.2 ^a^	45.5 ± 0.4 ^a^
Fertilization (F)	*	**	**
S_CF100_	27.5 ± 0.3 ^c^	11.8 ± 0.1 ^c^	34.3 ± 0.4 ^c^
S_CF75_ + F_CF25_	27.1 ± 0.2 ^c^	11.5 ± 0.1 ^c^	33.4 ± 0.3 ^c^
S_CF75_ + F_CF25_ + C_L_	34.9 ± 0.3 ^b^	17.5 ± 0.2 ^b^	43.9 ± 0.4 ^b^
S_CF75_ + F_CF25_ + C_L_B	43.1 ± 0.4 ^a^	21.8 ± 0.2 ^a^	54.5 ± 0.4 ^a^
S × F	*	**	**
2019 × S_CF100_	25.6 ± 0.2 ^d^	10.4 ± 0.1 ^e^	31.2 ± 0.3 ^d^
2019 × S_CF75_ + F_CF25_	24.8 ± 0.2 ^d^	10.0 ± 0.1 ^e^	29.9 ± 0.3 ^d^
2019 × S_CF75_ + F_CF25_ + C_L_	31.4 ± 0.2 ^c^	15.3 ± 0.1 ^c^	39.4 ± 0.3 ^c^
2019 × S_CF75_ + F_CF25_ + C_L_B	39.5 ± 0.3 ^b^	19.4 ± 0.2 ^b^	49.3 ± 0.4 ^b^
2020 × S_CF100_	29.4 ± 0.3 ^c^	13.2 ± 0.1 ^d^	37.4 ± 0.4 ^c^
2020 × S_CF75_ + F_CF25_	29.3 ± 0.2 ^c^	12.9 ± 0.1 ^d^	36.8 ± 0.3 ^c^
2020 × S_CF75_ + F_CF25_ + C_L_	38.4 ± 0.3 ^b^	19.6 ± 0.2 ^b^	48.3 ± 0.4 ^b^
2020 × S_CF75_ + F_CF25_ + C_L_B	46.6 ± 0.4 ^a^	24.1 ± 0.2 ^a^	59.6 ± 0.4 ^a^
**Source of Variation**	**Available Micronutrient Contents (mg kg^−1^ Soil)**		
	**Available Fe**	**Available Mn**	**Available Zn**
Season (S)	*	*	*
2019	4.61 ± 0.26 ^b^	3.13 ± 0.16 ^b^	2.22 ± 0.14 ^b^
2020	6.08 ± 0.37 ^a^	4.56 ± 0.24 ^a^	3.16 ± 0.16 ^a^
Fertilization (F)	**	*	*
S_CF100_	4.09 ± 0.25 ^c^	3.15 ± 0.16 ^c^	2.28 ± 0.13 ^c^
S_CF75_ + F_CF25_	4.11 ± 0.27 ^c^	3.10 ± 0.16 ^c^	2.24 ± 0.11 ^c^
S_CF75_ + F_CF25_ + C_L_	5.70 ± 0.33 ^b^	4.06 ± 0.21 ^b^	2.80 ± 0.17 ^b^
S_CF75_ + F_CF25_ + C_L_B	7.47 ± 0.41 ^a^	5.08 ± 0.26 ^a^	3.44 ± 0.18 ^a^
S × F	**	*	*
2019 × S_CF100_	3.52 ± 0.22 ^d^	2.28 ± 0.14 ^d^	1.74 ± 0.10 ^d^
2019 × S_CF75_ + F_CF25_	3.64 ± 0.23 ^d^	2.22 ± 0.12 ^d^	1.70 ± 0.08 ^d^
2019 × S_CF75_ + F_CF25_ + C_L_	4.48 ± 0.25 ^c^	3.55 ± 0.16 ^c^	2.38 ± 0.18 ^c^
2019 × S_CF75_ + F_CF25_ + C_L_B	6.79 ± 0.34 ^b^	4.48 ± 0.22 ^b^	3.04 ± 0.18 ^b^
2020 × S_CF100_	4.66 ± 0.27 ^c^	4.02 ± 0.18 ^bc^	2.82 ± 0.16 ^c^
2020 × S_CF75_ + F_CF25_	4.58 ± 0.31 ^c^	3.98 ± 0.20 ^bc^	2.78 ± 0.14 ^c^
2020 × S_CF75_ + F_CF25_ + C_L_	6.92 ± 0.40 ^b^	4.57 ± 0.26 ^b^	3.21 ± 0.16 ^b^
2020 × S_CF75_ + F_CF25_ + C_L_B	8.15 ± 0.48 ^a^	5.68 ± 0.30 ^a^	3.84 ± 0.18 ^a^

** and * indicate, respectively, differences at *p* ≤ 0.05 and *p* ≤ 0.01 probability level, respectively, and ns indicates not significant difference. Mean values with different letters in each column are significant (*p* ≤ 0.05). S_CF100_ = chemical fertilizers applied to the soil at full recommended NPK doses, S**_CF75_** = chemical fertilizers applied to the soil at 75% of recommended NPK doses, F**_CF25_** = chemical fertilizers applied as foliar sprays at 25% of recommended NPK doses, C**_L_** = 1.2 kg leguminous compost m^−2^, and C**_L_**B = biocompost; leguminous compost (1.2 kg per m^−2^) containing bacteria.

**Table 4:** Response of growth and productivity of *Phaseolus vulgaris* plants to growing season (2019 and 2020) and different fertilization strategies applied to virgin sandy soil.

Source of Variation	Growth Parameters		Yield Parameters	
	Shoot FW (g Plant^−1^)	Shoot DW (g Plant^−1^)	Green Pod Weight (ton ha^−1^)	Dry Seed Weight (ton ha^−1^)
Season (S)	*	*	*	*
2019	18.3 ± 1.7 ^b^	2.25 ± 2.1 ^b^	2.62 ± 0.28 ^b^	0.67 ± 0.07 ^b^
2020	22.1 ± 2.2 ^a^	2.60 ± 2.6 ^a^	3.00 ± 0.30 ^a^	0.86 ± 0.09 ^a^
Fertilization (F)	**	*	*	**
S_CF100_	15.3 ± 1.4 ^d^	1.95 ± 2.1 ^d^	2.16 ± 0.23 ^d^	0.52 ± 0.06 ^d^
S_CF75_ + F_CF25_	17.9 ± 1.9 ^c^	2.22 ± 2.1 ^c^	2.62 ± 0.26 ^c^	0.63 ± 0.07 ^c^
S_CF75_ + F_CF25_ + C_L_	21.6 ± 2.1 ^b^	2.59 ± 2.6 ^b^	3.07 ± 0.32 ^b^	0.83 ± 0.08 ^b^
S_CF75_ + F_CF25_ + C_L_B	26.1 ± 2.5 ^a^	2.95 ± 2.8 ^a^	3.39 ± 0.34 ^a^	1.09 ± 0.11 ^a^
S × F	**	*	*	**
2019 × S_CF100_	14.2 ± 1.3 ^e^	1.79 ± 1.8 ^e^	1.96 ± 0.21 ^e^	0.46 ± 0.05 ^e^
2019 × S_CF75_ + F_CF25_	16.6 ± 1.7 ^d^	2.09 ± 1.9 ^d^	2.41 ± 0.25 ^d^	0.55 ± 0.05 ^d^
2019 × S_CF75_ + F_CF25_ + C_L_	19.0 ± 1.8 ^c^	2.36 ± 2.2 ^c^	2.90 ± 0.31 ^c^	0.72 ± 0.07 ^c^
2019 × S_CF75_ + F_CF25_ + C_L_B	23.4 ± 2.1 ^b^	2.75 ± 2.5 ^b^	3.19 ± 0.34 ^b^	0.96 ± 0.10 ^b^
2020 × S_CF100_	16.4 ± 1.5 ^d^	2.11 ± 2.3 ^d^	2.36 ± 0.25 ^d^	0.57 ± 0.07 ^d^
2020 × S_CF75_ + F_CF25_	19.1 ± 2.0 ^c^	2.34 ± 2.3 ^c^	2.83 ± 0.27 ^c^	0.69 ± 0.08 ^c^
2020 × S_CF75_ + F_CF25_ + C_L_	24.2 ± 2.3 ^b^	2.81 ± 2.9 ^b^	3.24 ± 0.32 ^b^	0.94 ± 0.08 ^b^
2020 × S_CF75_ + F_CF25_ + C_L_B	28.7 ± 2.8 ^a^	3.14 ± 3.0 ^a^	3.58 ± 0.34 ^a^	1.22 ± 0.11 ^a^

** and * indicate, respectively, differences at *p* ≤ 0.05 and *p* ≤ 0.01 probability level, respectively. Mean values with different letters in each column are significant (*p* ≤ 0.05). S_CF100_ = chemical fertilizers applied to the soil at full recommended NPK doses, S**_CF75_** = chemical fertilizers applied to the soil at 75% of recommended NPK doses, F**_CF25_** = chemical fertilizers applied as foliar sprays at 25% of recommended NPK doses, C**_L_** = 1.2 kg leguminous compost m^−2^, and C**_L_**B = biocompost; leguminous compost (1.2 kg m^−2^) containing bacteria.

**Table 5:** Response of nutritional contents of *Phaseolus vulgaris* plants to growing season (2019 and 2020) and different fertilization strategies applied to virgin sandy soil.

Source of Variation	Macronutrient Contents (mg g^−1^ DW)			Micronutrient Contents (mg kg^−1^ DW)		
	N	P	K	Fe	Mn	Zn
Season (S)	*	*	*	*	*	*
2019	21.1 ± 0.5 ^b^	1.93 ± 0.05 ^b^	24.0 ± 0.6 ^b^	274 ± 14 ^b^	180 ± 7 ^b^	126 ± 4 ^b^
2020	25.3 ± 0.8 ^a^	2.42 ± 0.07 ^a^	27.8 ± 0.8 ^a^	322 ± 17 ^a^	223 ± 9 ^a^	151 ± 5 ^a^
Fertilization (F)	*	**	*	*	**	**
S_CF100_	16.9 ± 0.5 ^d^	1.47 ± 0.04 ^d^	19.4 ± 0.5 ^d^	221 ± 12 ^d^	144 ± 6 ^d^	100 ± 4 ^d^
S_CF75_ + F_CF25_	20.9 ± 0.6 ^c^	1.89 ± 0.05 ^c^	23.6 ± 0.6 ^c^	265 ± 14 ^c^	176 ± 7 ^c^	127 ± 5 ^c^
S_CF75_ + F_CF25_ + C_L_	25.6 ± 0.7 ^b^	2.43 ± 0.07 ^b^	28.1 ± 0.7 ^b^	327 ± 17 ^b^	222 ± 9 ^b^	152 ± 5 ^b^
S_CF75_ + F_CF25_ + C_L_B	29.3 ± 0.8 ^a^	2.92 ± 0.09 ^a^	32.6 ± 0.9 ^a^	378 ± 21 ^a^	264 ± 10 ^a^	176 ± 6 ^a^
S × F	*	**	*	*	**	**
2019 × S_CF100_	15.2 ± 0.4 ^e^	1.22 ± 0.03 ^e^	17.8 ± 0.3 ^e^	198 ± 10 ^e^	128 ± 5 ^e^	89 ± 3 ^e^
2019 × S_CF75_ + F_CF25_	18.4 ± 0.5 ^d^	1.68 ± 0.04 ^d^	21.1 ± 0.5 ^d^	239 ± 12 ^d^	156 ± 6 ^d^	112 ± 4 ^d^
2019 × S_CF75_ + F_CF25_ + C_L_	23.1 ± 0.5 ^c^	2.14 ± 0.06 ^c^	26.4 ± 0.6 ^c^	298 ± 15 ^c^	192 ± 8 ^c^	136 ± 4 ^c^
2019 × S_CF75_ + F_CF25_ + C_L_B	27.6 ± 0.6 ^b^	2.67 ± 0.08 ^b^	30.6 ± 0.8 ^b^	360 ± 20 ^b^	244 ± 9 ^b^	165 ± 5 ^b^
2020 × S_CF100_	18.6 ± 0.6 ^d^	1.72 ± 0.05 ^d^	20.9 ± 0.6 ^d^	244 ± 14 ^d^	160 ± 7 ^d^	110 ± 4 ^d^
2020 × S_CF75_ + F_CF25_	23.4 ± 0.7 ^c^	2.10 ± 0.05 ^c^	26.0 ± 0.6 ^c^	290 ± 16 ^c^	196 ± 7 ^c^	141 ± 5 ^c^
2020 × S_CF75_ + F_CF25_ + C_L_	28.0 ± 0.9 ^b^	2.71 ± 0.07 ^b^	29.8 ± 0.8 ^b^	356 ± 18 ^b^	251 ± 9 ^b^	168 ± 5 ^b^
2020 × S_CF75_ + F_CF25_ + C_L_B	31.0 ± 0.9 ^a^	3.16 ± 0.09 ^a^	34.6 ± 1.0 ^a^	396 ± 20 ^a^	284 ± 11 ^a^	186 ± 7 ^a^

** and * indicate, respectively, differences at *p* ≤ 0.05 and *p* ≤ 0.01 probability level, respectively. Mean values with different letters in each column are significant (*p* ≤ 0.05). S_CF100_ = chemical fertilizers applied to the soil at full recommended NPK doses, S**_CF75_** = chemical fertilizers applied to the soil at 75% of recommended NPK doses, F**_CF25_** = chemical fertilizers applied as foliar sprays at 25% of recommended NPK doses, C**_L_** = 1.2 kg leguminous compost m^−2^, and C**_L_**B = biocompost; leguminous compost (1.2 kg m^−2^) containing bacteria.

**Table 6:** Response of photosynthetic parameters, relative water content, and osmoprotectant contents of *Phaseolus vulgaris* plants to growing season (2019 and 2020) and different fertilization strategies applied to virgin sandy soil.

Source of Variation	Photosynthetic Parameters			Leaf Relative Water Content (RWC) and Osmoprotectant Contents		
	TChl Content (g kg^‒1^ FW)	Fv/Fm	PI (%)	RWC (%)	Proline (μg g^−1^ DW)	TS Sugars (mg g^−1^ DW)
Season (S)	*	*	*	*	*	*
2019	1.76 ± 0.03 ^b^	0.77 ± 0.02 ^b^	13.7 ± 0.3 ^b^	65.0 ± 2.3 ^b^	160 ± 5 ^b^	18.0 ± 0.4 ^b^
2020	2.14 ± 0.04 ^a^	0.83 ± 0.03 ^a^	15.9 ± 0.4 ^a^	73.8 ± 2.5 ^a^	185 ± 6 ^a^	22.4 ± 0.5 ^a^
Fertilization (F)	**	*	*	*	**	**
S_CF100_	1.30 ± 0.03 ^d^	0.71 ± 0.02 ^d^	11.5 ± 0.3 ^d^	56.7 ± 2.1 ^d^	136 ± 4 ^d^	14.2 ± 0.4 ^d^
S_CF75_ + F_CF25_	1.69 ± 0.03 ^c^	0.76 ± 0.02 ^c^	13.4 ± 0.4 ^c^	64.5 ± 2.3 ^c^	163 ± 5 ^c^	18.0 ± 0.5 ^c^
S_CF75_ + F_CF25_ + C_L_	2.17 ± 0.04 ^b^	0.83 ± 0.03 ^b^	15.9 ± 0.4 ^b^	74.0 ± 2.5 ^b^	183 ± 7 ^b^	22.1 ± 0.5 ^b^
S_CF75_ + F_CF25_ + C_L_B	2.65 ± 0.05 ^a^	0.89 ± 0.03 ^a^	18.5 ± 0.4 ^a^	82.3 ± 2.9 ^a^	205 ± 7 ^a^	26.4 ± 0.6 ^a^
S × F	**	*	*	*	**	**
2019 × S_CF100_	1.12 ± 0.02 ^e^	0.68 ± 0.01 ^e^	10.4 ± 0.2 ^f^	52.4 ± 1.9 ^d^	125 ± 3 ^d^	12.4 ± 0.3 ^e^
2019 × S_CF75_ + F_CF25_	1.52 ± 0.02 ^d^	0.73 ± 0.02 ^d^	12.4 ± 0.3 ^e^	60.2 ± 2.1 ^c^	148 ± 4 ^d^	15.8 ± 0.4 ^d^
2019 × S_CF75_ + F_CF25_ + C_L_	1.97 ± 0.03 ^c^	0.79 ± 0.02 ^c^	14.9 ± 0.3 ^c^	69.6 ± 2.4 ^b^	176 ± 6 ^c^	19.6 ± 0.4 ^c^
2019 × S_CF75_ + F_CF25_ + C_L_B	2.44 ± 0.04 ^b^	0.86 ± 0.02 ^b^	17.2 ± 0.4 ^b^	77.8 ± 2.9 ^a^	192 ± 6 ^bc^	24.0 ± 0.5 ^b^
2020 × S_CF100_	1.48 ± 0.03 ^de^	0.74 ± 0.02 ^d^	12.6 ± 0.3 ^de^	61.0 ± 2.2 ^c^	147 ± 5 ^d^	16.0 ± 0.4 ^d^
2020 × S_CF75_ + F_CF25_	1.86 ± 0.03 ^cd^	0.79 ± 0.02 ^c^	14.4 ± 0.4 ^cd^	68.8 ± 2.5 ^b^	178 ± 5 ^c^	20.2 ± 0.5 ^c^
2020 × S_CF75_ + F_CF25_ + C_L_	2.37 ± 0.04 ^b^	0.87 ± 0.03 ^b^	16.9 ± 0.4 ^b^	78.4 ± 2.6 ^a^	196 ± 7 ^ab^	24.6 ± 0.5 ^b^
2020 × S_CF75_ + F_CF25_ + C_L_B	2.86 ± 0.05 ^a^	0.92 ± 0.03 ^a^	19.8 ± 0.4 ^a^	86.8 ± 2.8 ^a^	218 ± 7 ^a^	28.8 ± 0.6 ^a^

** and * indicate, respectively, differences at *p* ≤ 0.05 and *p* ≤ 0.01 probability level, respectively. Mean values with different letters in each column are significant (*p* ≤ 0.05). TChl = total chlorophylls, Fv/Fm = chlorophyll fluorescence, PI = performance index, S_CF100_ = chemical fertilizers applied to the soil at full recommended NPK doses, S**_CF75_** = chemical fertilizers applied to the soil at 75% of recommended NPK doses, F**_CF25_** = chemical fertilizers applied as foliar sprays at 25% of recommended NPK doses, C**_L_** = 1.2 kg leguminous compost m^−2^, and C**_L_**B = biocompost; leguminous compost (1.2 kg m^−2^) containing bacteria.

## Data Availability

The data presented in this study are available upon request from the corresponding author.

## References

[1] Alshankiti A., Gill S. (2016). Integrated Plant Nutrient Management for Sandy Soil Using Chemical Fertilizers, Compost, Biochar and Biofertilizers—Case Study in UAE. J. Arid Land Stud..

[2] Pain C.F., Abdelfattah M.A. (2015). Landform evolution in the arid northern United Arab Emirates: Impacts of tectonics, sea level changes and climate. Catena.

[3] Al-Muaini A.H., Green S.R., Abou Dahr W.A., Al-Yamani W., Abdelfattah M.A., Pangilinan R., McCann I., Dakheel A., Abdullah A., Kennedy L. (2019). Sustainable Irrigation of Date Palms in the Hyper-Arid United Arab Emirates: A Review. Chron. Hortic..

[4] Ksiksi T.S., Trueman R., Abdelfattah M.A., Mousa M.T., Almarzouqi A.Y., Barahim S.A. (2019). Above and belowground carbon pools are affected by dominant floral species in hyper-arid environments. F1000Research.

[5] Abdelfattah M.A. (2013). Pedogenesis, land management and soil classification in hyper-arid environments: Results and implications from a case study in the United Arab Emirates. Soil Use Manag. J..

[6] Idowu O.A., Lorentz S.A., Annandale J.G., McCartney M.P., Jovanovic N.Z. (2008). Assessment of the Impact of Irrigation with Low-quality Mine Water on Virgin and Rehabilitated Soils in the Upper Olifants Basin. Mine Water Environ..

[7] Wilson I.G. (1973). Ergs. Sediment. Geol..

[8] Fryberger S.G., McKee E.D. (1979). Dune forms and wind regime. A Study of Global Sand Seas, U.S. Geological Survey Professional Paper 1052.

[9] Thomas D.S.G. (1997). Arid Zone Geomorphology: Process, Forms and Change in Drylands.

[10] Pye K., Tsoar H. (1990). Aeolian Sand and Sand Dunes.

[11] Bubenzer O., Embabi N.S., Ashour M.M. (2020). Sand Seas and Dune Fields of Egypt. Geosci.

[12] Brataševec K., Sivilotti P., Vodopivec B.M. (2013). Soil and foliar fertilization affects mineral contents in Vitis vinifera L. cv. ‘rebula’ leaves. J. Soil Sci. Plant Nutr..

[13] Fregoni M. (1998). Viticoltura di Qualità.

[14] Kannan S., Lichtfouse E. (2010). Foliar Fertilization for Sustainable Crop Production. Genetic Engineering, Biofertilization, Soil Quality and Organic Farming.

[15] Tejada M., Gonzalez J.L. (2004). Effects off foliar application of a byproduct of the two-step olive oil mill process on rice yield. Europ. J. Agron..

[16] Kaya C., Higgs D. (2002). Response of tomato (*Lycopersicon esculentum* L.) cultivars to foliar application of zinc when grown in sand culture at low zinc. Sci. Hortic..

[17] Jamal Z., Hamayun M., Ahmed N., Chaudhary M.F. (2006). Effect of soil and foliar application of different concentrations of NPK and foliar application of (NH_4_)_2_SO_4_ on different yield parameters in wheat. Asian J. Agron..

[18] Dewdar M.D.H., Rady M.M. (2013). Influence of soil and foliar applications of potassium fertilization on growth, yield and fiber quality traits in two *Gossypium barbadense* L. varieties. Afr. J. Agric. Res..

[19] Basavaraj P., Chetan H.T. (2018). Folair fertilization of nutrients. Marumegh.

[20] Rady M.M. (2011). Effects on growth, yield, and fruit quality in tomato (Lycopersicon esculentum Mill.) using a mixture of potassium humate and farmyard manure as an alternative to mineral-N fertiliser. J. Hortic. Sci. Biotechnol..

[21] Rady M.M., Semida W.M., Hemida K.A., Abdelhamid M.T. (2016). The effect of compost on growth and yield of Phaseolus vulgaris plants grown under saline soil. Int. J. Recycl. Org. Waste Agric..

[22] Alharby H.F., Al-Zahrani H.S., Hakeem K.R., Alsamadany H., Desoky E.-S.M., Rady M.M. (2021). Silymarin-Enriched Biostimulant Foliar Application Minimizes the Toxicity of Cadmium in Maize by Suppressing Oxidative Stress and Elevating Antioxidant Gene Expression. Biomolecules.

[23] Kusparwanti T.R., Eliyatiningsih, Wardana R. (2020). Application Legume Compost with Bio-Activator Trichoderma sp as Inorganic Fertilizer Substitution in Sweet Corn (*Zea mays* L. Saccharata) Cultivation. IOP Conf. Series. Earth Environ. Sci..

[24] Bamagoos A.A., Alharby H.F., Belal E.E., Khalaf A.E.A., Abdelfattah M.A., Rady M.M., Ali E.F., Mersal G.A.M. (2021). Phosphate-Solubilizing Bacteria as a Panacea to Alleviate Stress Effects of High Soil CaCO_3_ Content in Phaseolus vulgaris with Special Reference to P-Releasing Enzymes. Sustainability.

[25] Giller K.E., McDonagh J.F., Cadisch G., Syers P.K., Rimmer D.L. (1994). Can biological nitrogen fixation sustain agriculture in the tropics?. Soil Science and Sustainable Land Management in the Tropics.

[26] Kumwenda J.D.T., Waddington S.R., Snapp S.S., Jones R.B., Blackie M.J. (1995). Soil Fertility Managementfor Smallholder Maize-Based Cropping Systems of Southern Afnca: A Review. Network Working Paper No. 1. Soil Fertility Network for Maize-Based Cropping Systems in Countries of Southern Africa.

[27] Cayuela M.L., Mondini C., Insam H., Sinicco T., Whittle F.I. (2009). Plant and animal wastes composting: Effects of the N source on process performance. Bioresour. Technol..

[28] Buttery B.R., Park S.l., Hume D.F.J. (1992). Potential for increasing nitrogen fixation in grain legumes. J. Plant Sci..

[29] Senjobi B.A. (2007). Comparative Assessment of the Effect of Land Use and Land Type on Soil Degradation and Agricultural Productivity in Ogun State, Nigeria. Ph.D. Thesis.

[30] Page A.I., Miller R.H., Keeny D.R. (1982). Methods of soil analysis. Part II. Chemical and Microbiological Methods.

[31] Klute A., Dirksen C. (1986). Hydraulic conductivity and diffusivity. Laboratory methods. Methods of Soil Analysis-Part 1. Phys. Mineral. Methods.

[32] OECD (2016). “Common Bean (Phaseolus Vulgaris)”, in Safety Assessment of Transgenic Organisms in the Environment, Volume 6: OECD Consensus Documents.

[33] Carter M.R., Gregorich E.G. (2006). Soil Sampling and Methods of Analysis.

[34] Hope C.F.A., Burns R.G. (1987). Activity, origins and location of cellulases in a silt loam soil. Biol. Fertil. Soils.

[35] Schinner F., von Mersi W. (1990). Xylanase-, CM-cellulase- and invertase activity in soil: An improved method. Soil Biol. Biochem..

[36] Miller G.L. (1972). Use of dinitrosalicylic acid reagent for the determination of glucose. Anal. Chem..

[37] Kandeler E., Gerber H. (1988). Short-term assay of soil urease activity using colorimetric determination of ammonium. Biol. Fertil. Soils.

[38] Johnson J.L., Temple K.L. (1964). Some variables affecting the measurement of “catalase activity” in soil. Soil Sci. Soc. Am. J..

[39] Stepniewska Z., Wolinska A., Ziomek J. (2009). Response of soil catalase activity to chromium contamination. J. Environ. Sci..

[40] King E.J. (1951). Micro-Analysis in Medical Biochemistry.

[41] Olsen S.R. (1954). Estimation of Available Phosphorus in Soils by Extraction with Sodium Bicarbonate.

[42] Chapman H.D., Norman A.G. (1965). Cation-Exchange capacity. Methods of Soil Analysis: Part 2 Chemical and Microbiological Properties.

[43] Arnon D.I. (1949). Copper enzymes in isolated chloroplasts, polyphenoxidase in Beta vulgaris. Plant Physiol..

[44] Li P.M., Cai R.G., Gao H.Y., Peng T., Wang Z.L. (2007). Partitioning of excitation energy in two wheat cultivars with different grain protein contents grown under three nitrogen applications in the field. Physiol. Plant..

[45] Maxwell K., Johnson G.N. (2000). Chlorophyll fluorescence—A practical guide. J. Exp. Bot..

[46] Clark A.J., Landolt W., Bucher J.B., Strasser R.J. (2000). Beech (*Fagus sylvatica*) response to ozone exposure assessed with a chlorophyll a fluorescence performance index. Environ. Pollut..

[47] Osman A.S., Rady M.M. (2014). Ameliorative effects of sulphur and humic acid on the growth, antioxidant levels, and yields of pea (*Pisum sativum* L.) plants grown in reclaimed saline soil. J. Hortic. Sci. Biotechnol..

[48] Irigoyen J.J., Emerich D.W., Sanchez-Diaz M. (1992). Water stress induced changes in the concentrations of proline and total soluble sugars in nodulated alfalfa (Medicago sativa) plants. Plant Physiol..

[49] Bates L.S., Waldren R.P., Teare I.D. (1973). Rapid determination of free proline for water stress studies. Plant Soil.

[50] Griffth O.W. (1980). Determination of glutathione and glutathione disulfide using glutathione reductase and 2 vinyl pyridine. Anal. Biochem..

[51] Mukherjee S.P., Choudhari M.A. (1983). Implications of water stress induced changes in the levels of endogenous ascorbic acid and hydrogen peroxide in Vigna seedlings. Physiol. Plant..

[52] Giannopolitis C.N., Ries S.K. (1977). Superoxide dismutases. I. Occurrence in higher plants. Plant Physiol..

[53] Aebi H. (1984). Catalase in vitro. Methods Enzymol..

[54] Putter J., Bergmeyer H.U. (1974). Peroxidase. Methods of Enzymatic Analysis.

[55] Bradford M.M. (1976). A rapid and sensitive for the quantitation of microgram quantities of protein utilizing the principle of protein-dye binding. Anal. Biochem..

[56] Shapiro S.S., Wilk M.B. (1965). An Analysis of Variance Test for Normality (Complete Samples). Biometrika.

[57] Razali N., Wahi Y.B. (2011). Power comparisons of Shapiro–Wilk, Kolmogorov–Smirnov, Lilliefors and Anderson–Darling tests. J. Stat. Model. Anal..

[58] Malhi G.S., Kaur M., Kaushik P. (2021). Impact of climate change on agriculture and its mitigation strategies: A review. Sustainability.

[59] Abdelfattah M.A., Behnassi M., Mirza B.B., El Haiba M., Reed M. (2021). Climate Change Impacts on Water Resources and Food Security in Egypt and Possible Adaptive Measures—A review. Climate Change, Food Security, and Sustainable Smart Agriculture.

[60] Shahid S.A., Abdelfattah M.A., Taha F.K. (2013). Developments in Soil Salinity Assessment and Reclamation, Innovative Thinking and Use of Marginal Soil and Water Resources in Irrigated Agriculture.

[61] Shahid S.A., Taha F.K., Abdelfattah M.A. (2013). Developments in Soil Classification, Land Use Planning and Policy Implications, Innovative Thinking of Soil Inventory for Land Use Planning and Management of Land Resources.

[62] Shahid S.A., Abdelfattah M.A., Wilson M.A., Kelley J.A., Chiaretti J.V. (2014). United Arab Emirates Keys to Soil Taxonomy.

[63] Pain C.F., Abdelfattah M.A., Shahid S.A., Ditzler C., Zinck J.A., Metternicht G., Bocco G., Del Valle H.F. (2016). Soil-landform relationships in the arid Northern United Arab Emirates. Geopedology, An Integration of Geomorphology and Pedology for Soil and Landscape Studies.

[64] Shahid S.A., Abdelfattah M.A., Mahmoudi H., Shahid S.A., Taha F.K., Abdelfattah M.A. (2013). Innovations in soil chemical analyses—New ECe and total ions relationship in Abu Dhabi Emirate soils. Developments in Soil Classification, Land Use Planning and Policy Implications, Innovative Thinking of Soil Inventory for Land Use Planning and Management of Land Resources.

[65] Shahid S.A., Abdelfattah M.A., Othman Y., Kumar A., Taha F.K., Kelley J.A., Wilson M.A., Shahid S.A., Taha F.K., Abdelfattah M.A. (2013). Innovative Thinking for Sustainable Use of Terrestrial Resources in Abu Dhabi Emirate Through Scientific Soil Inventory and Policy Development. Developments in Soil Classification, Land Use Planning and Policy Implications, Innovative Thinking of Soil Inventory for Land Use Planning and Management of Land Resources.

[66] Shendi M.M., Abdelfattah M.A., Harbi A., Shahid S.A., Abdelfattah M.A., Taha F.K. (2013). Spatial monitoring of soil salinity and prospective conservation study for Sinnuris District soils, Fayoum, Egypt. Developments in Soil Salinity Assessment and Reclamation, Innovative Thinking and Use of Marginal Soil and Water Resources in Irrigated Agriculture.

[67] Kelley J.A., Wilson M.A., Abdelfattah M.A., Shahid S.A., Shahid S.A., Taha F.K., Abdelfattah M.A. (2013). Quality Assurance Standards: USDA Perspective of the Extensive Soil Survey of Abu Dhabi Emirate. Developments in Soil Classification, Land Use Planning and Policy Implications, Innovative Thinking of Soil Inventory for Land Use Planning and Management of Land Resources.

[68] King P., Grealish G., Shahid S.A., Abdelfattah M.A., Shahid S.A., Taha F.K., Abdelfattah M.A. (2013). Land evaluation interpretations—Soil Survey of Abu Dhabi Emirate. Developments in Soil Classification, Land Use Planning and Policy Implications, Innovative Thinking of Soil Inventory for Land Use Planning and Management of Land Resources.

[69] Wilson M.A., Shahid S.A., Abdelfattah M.A., Kelley J.A., Thomas J.E., Shahid S.A., Taha F.K., Abdelfattah M.A. (2013). Anhydrite formation on the coastal sabkha of Abu Dhabi, United Arab Emirates. Developments in Soil Classification, Land Use Planning and Policy Implications, Innovative Thinking of Soil Inventory for Land Use Planning and Management of Land Resources.

[70] Verboom W.H., Pate J.S., Abdelfattah M.A., Shahid S.A., Shahid S.A., Taha F.K., Abdelfattah M.A. (2013). Effects of plants on soil forming processes: Case studies from arid environments. Developments in Soil Classification, Land Use Planning and Policy Implications, Innovative Thinking of Soil Inventory for Land Use Planning and Management of Land Resources.

[71] Clark S. (2020). Organic farming and climate change: The need for innovation: An opinion. Sustainability.

[72] Gransee A., Fuhrs H. (2013). Magnesium mobility in soils as a challenge for soil and plant analysis magnesium fertilization and root uptake under adverse growth conditions. Plant Soil.

[73] Rehman H., Alharby H.F., Alzahrani Y., Rady M.M. (2018). Magnesium and organic biostimulant integrative application induces physiological and biochemical changes in sunflower plants and its harvested progeny on sandy soil. Plant Physiol. Biochem..

[74] Shahid S.A., Taha F.K., Ismail S., Al Dakheel A., Abdelfattah M.A., Behnassi M., Shahid S.A., D’Silva J. (2011). Turning Adversity into an Advantage for Food Security Through Improving Soil Quality and Providing Production Systems for Marginal Saline Lands: ICBA Perspectives and Approaches. Sustainable Agriculture Development, Recent Approaches in Resources Management and Environmentally-Balanced Production Enhancement.

[75] Buragohain S., Sarma B., Nath D.J., Gogoi N., Meena R.S., Lal R. (2018). Effect of 10 years of biofertiliser use on soil quality and rice yield on an Inceptisol in Assam, India. Soil Res..

[76] Belal E.E., El Sowfy D.M., Rady M.M. (2019). Integrative soil application of humic acid and sulfur improves saline calcareous soil properties and barley plant performance. Commun. Soil Sci. Plant Anal..

[77] Manirakiza N., Şeker C. (2020). Effects of compost and biochar amendments on soil fertility and crop growth in a calcareous soil. J. Plant Nutr..

[78] Rady M.M., El-Shewy A.A., Seif El-Yazal M.A., Abd El-Gawwad I.F.M. (2020). Integrative application of soil P-solubilizing bacteria and foliar nano p improves Phaseolus vulgaris plant performance and antioxidative defense system components under calcareous soil conditions. J. Soil Sci. Plant Nutr..

[79] Sinha S.K., Kumar V., Jha C.K. (2017). Effect of Integrated use of Bio-Compost and Nitrogen on Productivity and Soil Properties of Sugarcane Plant–Ratoon System in Calcareous Soil. SugarTech.

[80] Wu S.C., Cao Z.H., Li Z.G., Cheung K.C., Wong M.H. (2005). Effects of biofertilizer containing N-fixer, P and K solubilizers and AM fungi on maize growth: A greenhouse trial. Geoderma.

[81] Valarini P.J., Díaz M.C., Gascó J.M., Guerrero F., Tokeshi H. (2003). Assessment of soil properties by organic matter and EM-Microorganisms incorporation. Rev. Bras. De Ciência Do Solo.

[82] Fischer D., Glaser B., Kumar S. (2012). Synergisms between compost and biochar for sustainable soil amelioration. Management of Organic Waste.

[83] Aboukila E.F., Nassar I.N., Rashad M., Hafez M., Norton J.B. (2018). Reclamation of calcareous soil and improvement of squash growth using brewers’ spent grain and compost. J. Saudi Soc. Agric. Sci..

[84] Nath D.J., Ozha B., Barooah R.C., Borah D.K. (2012). Effect of integrated nutrient management on soil enzymes, microbial biomass carbon and bacterial populations under rice (*Oryza sativa*)–wheat (*Triticum aestivum*) sequence. Indian J. Agric. Sci..

[85] Macci C., Doni S., Peruzzi E., Masciandaro G., Mennone C., Ceccanti B. (2010). Almond tree and organic fertilization for soil quality improvement in southern Italy. J. Environ. Manag..

[86] Swarnalakshmi K., Prasanna R., Kumar A., Pattnaik S., Chakravarty K., Shivay Y.S., Singh R., Saxena A.K. (2013). Evaluating the influence of novel cyanobacterial bio filmed biofertilizers on soil fertility and plant nutrition in wheat. Euro. J. Soil Biol..

[87] Hamayun M., Khan S.A., Khan A.L., Shinwari Z.K., Ahmad N., Kim Y.H., Lee I.J. (2011). Effect of foliar and soil application of nitrogen, phosphorus and potassium on yield components of lentil. Pak. J. Bot..

[88] Rahman M.A., Rahman M.M., Begum M.F., Alam M.F. (2012). Effect of bio compost, cow dung compost and NPK fertilizers on growth, yield and yield components of chili. Int. J. Biosci..

[89] Sandoval A.P., Yáñez-Chávez L.G., Sánchez-Cohen I., Samaniego-Gaxiola J.A., Trejo-Calzada R. (2017). Hydrogel, biocompost and its effect on photosynthetic activity and production of forage maize (*Zea mays* L.) plants. Acta Agron..

[90] Sinclair T.R., Ludlow M.M. (1986). Influence of soil water supply on the plant water balance of four tropical grain legumes. Aust. J. Plant Physiol..

[91] Nusrat N., Shahbaz M., Perveen S. (2014). Modulation in growth, photosynthetic efficiency, activity of antioxidants and mineral ions by foliar application of glycinebetaine on pea (*Pisum sativum* L.) under salt stress. Acta Physiol. Plant..

[92] Rady M.M., Rehman H.U. (2016). Supplementing organic biostimulants into growing media enhances growth and nutrient uptake of tomato transplants. Sci. Hortic..

[93] Rady M.M., Elrys E.S., Abo El-Maati M.E., Desoky E.M. (2019). Interplaying roles of silicon and proline effectively improve salt and cadmium stress tolerance in *Phaseolus vulgaris* plant. Plant Physiol. Biochem..

[94] Semida W.M., Hemida K.A., Rady M.M. (2018). Sequenced ascorbate-proline-glutathione seed treatment elevates cadmium tolerance in cucumber transplants. Ecotoxicol. Environ. Saf..

[95] Alharby H.F., Alzahrani H.S., Alzahrani Y., Alsamadany H., Hakeem K.R., Rady M.M. (2021). Maize grain extract enriched with polyamines alleviates drought stress in Triticum aestivum through up-regulation of the ascorbate-glutathione cycle, glyoxalase system, and polyamine gene expression. Agronomy.

[96] Rady M.M., Desoky E.-S.M., Ahmed S.M., Majrashi A., Ali E.F., Arnaout S.M.A., Selem E. (2021). Foliar Nourishment with Nano-Selenium Dioxide Promotes Physiology, Biochemistry, Antioxidant Defenses, and Salt Tolerance in *Phaseolus vulgaris*. Plants.

[97] Rady M.M., Boriek S.H.K., Abd El-Mageed T.A., Seif El-Yazal M.A., Ali E.F., Hassan F.A.S., Abdelkhalik A. (2021). Exogenous Gibberellic Acid or Dilute Bee Honey Boosts Drought Stress Tolerance in *Vicia faba* by Rebalancing Osmoprotectants, Antioxidants, Nutrients, and Phytohormones. Plants.

[98] Desoky E.S., Mansour E., Ali M.M.A., Yasin M.A.T., Abdul-Hamid M.I.E., Rady M.M., Ali E.F. (2021). Exogenously used 24-epibrassinolide promotes drought tolerance in maize hybrids by improving plant and water productivity in an arid environment. Plants.

[99] Semida W.M., Abdelkhalik A., Mohamed G.F., Abd El-Mageed T.A., Abd El-Mageed S.A., Rady M.M., Ali E.F. (2021). Foliar Application of Zinc Oxide Nanoparticles Promotes Drought Stress Tolerance in Eggplant (*Solanum melongena* L.). Plants.

[100] Conde C., Delrot S., Geros H. (2008). Physiological, biochemical and molecular changes occurring during olive development and ripening. J. Plant Physiol..

